# The dynamics of the HIV epidemic among men who have sex with men (MSM) from 2005 to 2012 in Shenzhen, China

**DOI:** 10.1038/srep28703

**Published:** 2016-06-29

**Authors:** Jin Zhao, Lin Chen, Antoine Chaillon, Chenli Zheng, Wende Cai, Zhengrong Yang, Guilian Li, Yongxia Gan, Xiaohui Wang, Yihong Hu, Ping Zhong, Chiyu Zhang, Davey M. Smith

**Affiliations:** 1 Shenzhen Center for Disease Control and Prevention, Shenzhen, China; 2 University of California San Diego, La Jolla, California, USA; 3 Veterans Affairs Healthcare System San Diego, San Diego, California, USA; 4 Pathogen Diagnostic Center, Institut Pasteur of Shanghai, Chinese Academy of Sciences, Shanghai, China; 5 Shanghai Municipal Center for Disease Control and Prevention, Shanghai, China

## Abstract

HIV-1 epidemics among MSM are a major public health concern in China, especially in large cities. This study sought to better understand the dynamics of HIV molecular epidemiology among MSM in Shenzhen, a rapidly developing city with over 13.8 million people. HIV-1 pol sequences were obtained from 996 (53.5%) of 1862 HIV-infected MSM and 403(9.0%) of 4498 heterosexuals and injection drug users in Shenzhen, China from 2005-2012. Eight HIV-1 subtypes and some inter-subtype recombinants were identified among sampled MSM with CRF07_BC (39.1%) and CRF01_AE (35.1%) being the most predominant. From 2006 to 2012, the prevalence of CRF07_BC and CRF55_01B rapidly increased, while the prevalence of subtypes B and CRF01_AE gradually decreased. The genetic distances within CRF07_BC and CRF55_01B groups were significantly lower than within CRF01_AE and B groups. The vast majority (90.3%) of HIV-1 infected MSM in Shenzhen were migrants who came from 31 of the 34 provinces of China, and these migrants had significantly different HIV-1 subtype distributions from the local MSM. This study highlighted the importance of CRF07_BC and migrants in the changing HIV epidemic among MSM in China, and provides a molecular epidemiology framework for understanding how HIV-1 epidemics can change in large cities with diverse risk groups.

About 780,000 people live with HIV in China^1^, and sexual risk remains the most common mode of transmission. An important risk group is men who have sex with men (MSM)^2^, and currently China has over 21 million MSM^3^. The prevalence of HIV among Chinese MSM has rapidly increased from 0.9% in 2003 to 7.3% in 2013^2^. Another challenge for the expanding HIV epidemic among MSM is the large scale migrations of ‘floating’ individuals moving to large cities in the setting of fast and unbalanced economic development^4^,^5^,^6^,^7^,^8^. Overall, HIV prevalence among these migrants throughout China is also rapidly increasing, and is estimated to be 0.23% currently^9^,^10^. Such migration may fuel local epidemics^10^,^11^ and change the distribution of circulating HIV-1 subtypes^12^. The rapid increase of HIV prevalence among MSM and migrant populations, as well as a lack of effective intervention strategies for these groups, is a major challenge that China faces in the prevention and control of HIV^13^,^14^.

Shenzhen, located in Guangdong province in southern China, is a rapidly growing city with 13.8 million people currently. The majority of its population (81.8%) is ‘non-local residents’ who have household registration in other regions. These ‘non-local’ residents include ‘temporary’ residents (60%) and ‘floating’ individuals (21.8%)^15^. Temporary residents are individuals who have household registrations in other regions and have stayed in Shenzhen city more than six months, while ‘floating’ residents are individuals who have household registrations in other regions and have stayed in Shenzhen less than six months. Over 100,000 MSM are living in Shenzhen^16^,^17^ and the HIV-1 prevalence among them has rapidly increased from 0.2% in 2002 to 10.3% in 2011^17^,^18^. In this study, we sought to characterize the changing molecular epidemiology of the HIV epidemic among MSM and migrant populations in Shenzhen, China to elucidate possible opportunities for prevention.

## Results

### HIV prevalence among MSM in Shenzhen from 2005–2012

From 2005 to 2012, a total of 6,348,252 individuals in Shenzhen, China were screened for HIV, and 6,822 (0.11%) were found to be HIV-1 seropositive (data from the Shenzhen Center For Disease Control, HIV/AIDS Prevention and Control Division)^19^ Overall, HIV-1 prevalence rates were relatively stable in Shenzhen from 2005 to 2012 (0.09–0.13%). However, during this time the proportion of HIV-infected individuals reporting MSM risk among annual newly reported HIV cases increased from 3.9% in 2005 to 42.8% in 2012 (p < 0.01), and the overall proportion of annual newly reported HIV cases reporting MSM risk in Shenzhen was 27.3% over the study period. To better understand the HIV epidemic among MSM, around half (996/1862) of HIV-infected individuals reporting MSM risk were analyzed in this study.

### HIV-1 subtype distribution among MSM

Among 996 HIV-infected individuals reporting MSM risk, 8 HIV-1 subtypes, including B, C, CRF01_AE, CRF07_BC, CRF08_BC, CRF33_01B, CRF55_01B and CRF67_01B, as well as some unique recombinant forms (URFs) were identified. CRF07_BC (39.1%, 389/996) and CRF01_AE (35.1%, 350/996) were the most predominant subtypes, followed by CRF55_01B (12.7%, 126/996) and B (10.1%, 101/996). Subtypes C, CRF08_BC, CRF33_01B and CRF67_01B were rare with prevalence of 0.1%. In addition, 26 (2.6%) URF were identified (Supplementary Fig. S1).

**Figure 1:**
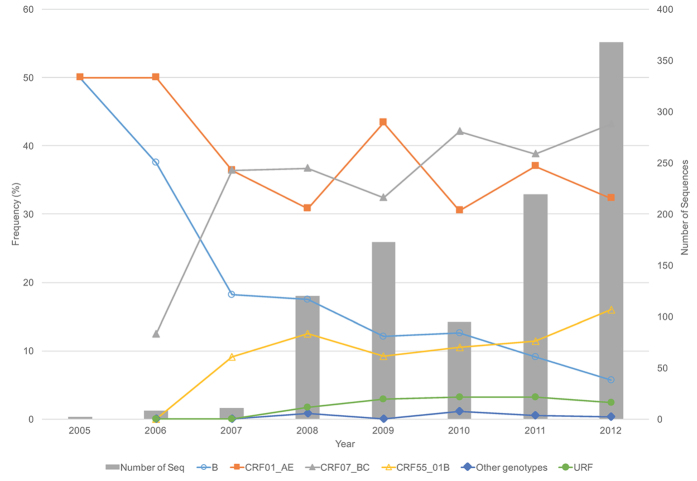
HIV-1 subtype distribution among individuals reporting MSM risk in Shenzhen from 2006 to 2012. Other subtypes include C, CRF08_BC, CRF33_01B, and CRF67_01B. The results in 2005 were not included in the figure because of only three sequences available.

From 2006 to 2012, the prevalence of HIV-1 subtypes B and CRF01_AE appeared to gradually decrease from 37.5% and 50.0% to 5.7% (*p* *<* *0.01*) and 32.3% (*p* *=* *0.19*), respectively (Fig. 1). In contrast, the prevalence of CRF07_BC and CRF55_01B rapidly increased from 12.5% and 0% in 2006 to 43.2% (*p* *=* *0.04*) and 16.0% (*p* *=* *0.03*) in 2012, respectively (Fig. 1). The prevalence of subtype CRF07_BC has exceeded the prevalence of subtype CRF01_AE among individuals with MSM risk in Shenzhen since 2008.

### Social-demographic characteristics of sampled MSM

The social-demographic characteristics of 996 individuals reporting MSM risk are summarized in Table 1. Their median age was 34.8 years old (range from 20 to 79 years old), and a vast majority (84.1%) were between 26–45 years old. Three-fourths (74.3%) of them reported being ‘single’, and 79.5% reported being ‘employed’. All but one had Chinese nationality, and 95.9% (955/996) were ethnically Han.

### Relationship between HIV subtype distribution and migration

Of HIV-1 infected individuals reporting MSM risk in Shenzhen, 90.3% were migrants, 60.7% (605/996) ‘temporary’ residents and 29.5% (294/996) ‘floating’ individuals. The remaining 9.7% were local permanent residents in Shenzhen (Table 1). The distribution of HIV-1 subtypes was similar between temporary resident and floating populations. Local residents had slightly higher prevalence of CRF07_BC and lower prevalence of CRF01_AE than temporary resident and floating populations (Supplementary Fig. S2). Four rare subtypes: C, CRF08_BC, CRF33_01B and CRF67_01B were only found among temporary residents.

**Figure 2:**
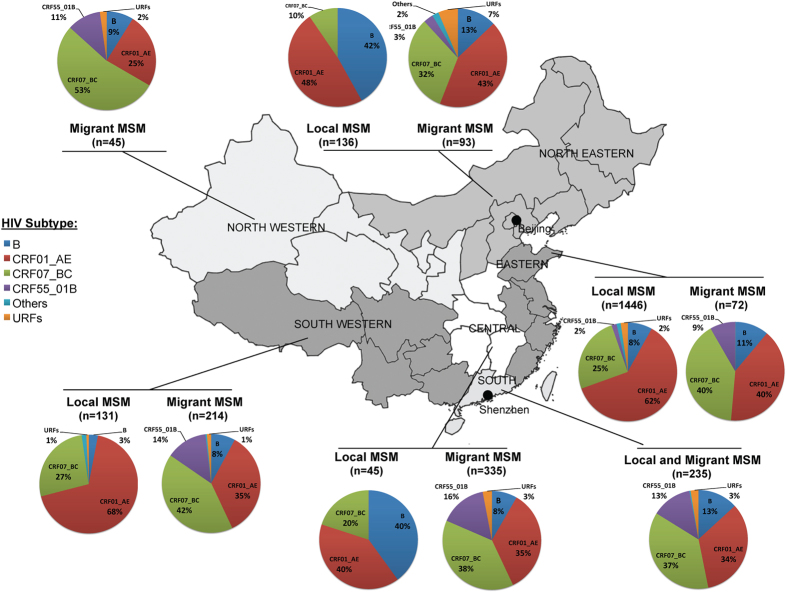
Geographic and HIV-1 subtype distributions of HIV-1-infected migrants reporting MSM risk in Shenzhen. The data of HIV-1 subtype information in different regions were obtained from references 12,26, 27, 28, 29, 30, 31, 32, 33, 34, and only the subtype results based on *pol* regions were used. Because the data was not available, no comparison was performed in Northwestern and Southern China. The information on the places of household registration of the migrants is shown in supplementary Table 2 in detail. Adapted from open access map: http://www.d-maps.com/carte.php?num_car=15275&lang=en.

HIV-1 infected migrants reporting MSM risk came from 31 of 34 provinces of China in 6 main geographic regions: i) Northwestern, ii) Northeastern, iii) Southwestern, iv) Southern, v) Eastern and vi) Central (Fig. 2 and Supplementary Table S1). The distributions of HIV-1 subtypes were not much different among these regions (Fig. 2), except migrants who came from Northwestern China had higher rate of CRF07_BC (*p* *<* *0.05*), while migrants from Northeastern China had slightly higher rate of CRF01_AE (*p* *=* *0.10*). Interestingly, when we compared the distribution of subtypes among migrants in Shenzhen with MSM from their home region, we found that the HIV subtype distributions among the migrants were significantly different from the MSM from their home region (p < 0.01, Pearson Chi-square test) (Fig. 2).

### HIV-1 subtype distributions of MSM and non-MSM risk groups

**Figure 3:**
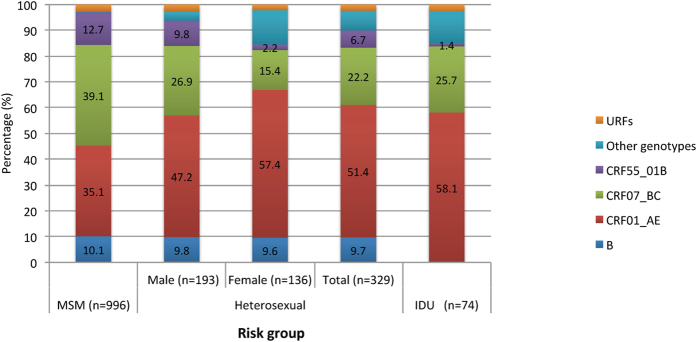
Comparison of HIV-1 genotype distributions between different risk groups.

We next compared the distribution of HIV subtypes among individuals reporting MSM risk versus those reporting heterosexual risk (HTS) and those reporting intravenous drug use risk (IDU). For this purpose, we randomly selected 403 individuals from non-MSM risk groups for sequencing: 329 HTS and 74 IDU. We found 10 HIV-1 subtypes among individuals reporting HTS risk and 5 subtypes among those reporting IDU risk. The prevalence of CRF01_AE was significantly higher among individuals reporting HTS (51.4%) and IDU (58.1%) risks than those reporting MSM risk (35.1%) (p < 0.01); however, the prevalence of CRF07_BC was significantly lower among the two non-MSM risk groups (HTS: 22.2% and IDU: 25.7%) than the MSM group (39.1%) (p < 0.01 and p = 0.02, respectively) (Fig. 3). Interestingly, the distribution among men reporting HTS risk was intermediate between men reporting MSM risk and female reporting HTS risk (Fig. 3). HIV-1 subtype B had similar prevalence among individuals reporting MSM and HTS risks, but it was not found among individuals reporting IDU risk (p < 0.01). CRF55_01B was significantly more prevalent among men reporting MSM (12.7%) and HTS (9.8%) risks than female reporting HTS risk (2.2%) (p < 0.01) and individuals reporting IDU risk (1.4%) (p = 0.02) (Fig. 3).

### Expansion of four main HIV-1 subtypes among sampled MSM

To estimate the expansion of the local epidemics, we evaluated the genetic distances within four main HIV-1 subtypes that were sampled The mean genetic distance for the CRF07_BC, CRF01_AE, CRF55_01B and B epidemics were 0.014, 0.039, 0.016 and 0.062, respectively. Overall, the genetic distances within CRF07_BC and CRF55_01B epidemics were significantly lower than the CRF01_AE and B epidemics (p < 0.01). This lower genetic distance could represent highly related and rapidly expanding transmission networks among MSM in Shenzhen. To evaluate this, we compared the genetic distance of all available subtype B, CRF01_AE, CRF07_BC and CRF08_BC *pol* sequences from China downloaded from the HIV LANL database^20^ and these subtype sequences from our study. Across all non-B subtypes, the mean genetic distance for LANL sequences were significantly higher than the genetic distance of sequences from our cohort (both p < 0.01, Supplementary Table S3).

## Discussion

This report has three important findings. First, the vast majority of HIV-1 infected individuals reporting MSM risk in Shenzhen were migrants who came from 31 of the 34 provinces of China, and HIV subtype distributions among these migrants in Shenzhen were significantly different than the subtype distribution among MSM living in the home region of these migrants (p < 0.01). Second, CRF07_BC had replaced CRF01_AE as the most predominant HIV-1 subtype circulating among MSM in Shenzhen. Third, the mean pairwise genetic distance within CRF07_BC was significantly lower than within CRF01_AE (supplementary table S3).

Migration contributes to the spread of HIV-1^21^, and is associated with an increased risk of HIV-1 infection in the rural population^8^,^10^,^11^. In China, uneven economic development in urban areas has led to large scale migration from rural areas to urban areas for better employment opportunities and living conditions. In particular, 245 million people comprised a floating population in China by the end of 2013, and majority of them were in large cities, especially in the eastern and southern regions of China^4^. This floating population had a higher prevalence of HIV than general population^10^,^11^ and the HIV prevalence among this population has increased gradually^9^. Sexual exposures were the primary risk factors associated with HIV infection for migrants^5^,^8^, and relative to other individuals in the floating population, migrant MSM were more at highest risk for HIV^7^,^22^,^23^. When these populations move between Shenzhen and their home regions, they may serve as a bridge between at-risk and non-risk populations^7^,^8^,^24^.

Urban areas may be particularly attractive to MSM, since large cities have relatively open culture and convenient sexual venues (bars, saunas, parks, sex clubs, etc.)^10^. Shenzhen has over 100,000 MSM, and vast majority (over 90%) of them are migrants^16^,^17^. HIV prevalence among MSM in Shenzhen was previously estimated to be 10.3%^17^,^18^, while this study found that individuals reporting MSM risk accounted for 27.3% of HIV positive individuals in Shenzhen between 2005–2012 Although we did increase our overall surveillance from 2008, including among local MSM, our surveillance always included considerable representation from MSM testing for HIV, and the prevalence greatly increased in this sampled population. This dramatic increase in Shenzhen has been previously documented and discussed in^17^,^18^. Additionally, 90.3% of the HIV-infected MSM were migrants (including floating population and temporary residents) coming from 31 of the 34 provinces of China (Fig. 2 and Supplementary Table S1).

This study identified eight HIV-1 subtypes, as well as some recombinants, among individuals reporting MSM risk in Shenzhen (Supplementary Fig. S1). Subtypes CRF07_BC, CRF01_AE, CRF55_01B and B were the most common, accounting for 39.1%, 35.1%, 12.7% and 10.1%, respectively. Distributions of these subtypes were similar among the Shenzhen local residents, temporary residents and the floating population (Supplementary Fig. S2). Although the migrants in Shenzhen came from different regions, they had similar distributions of HIV-1 CRF07BC and CRF01_AE, except those from Northwest China with higher of CRF07_BC and Northeast China with slightly higher of CRF01_AE (Fig. 2). Importantly, however, migrants in Shenzhen had significantly different HIV-1 subtype distributions from MSM still living in their home regions (p < 0.01)^12^,^25^,^26^,^27^,^28^,^29^,^30^,^31^,^32^,^33^ (Fig. 2). Given the differences in HIV subtype infections among migrant MSM and the prevalence of subtypes in their home regions, this might suggest that HIV infections in these migrant MSM were likely not entirely from their home regions. For example, these results suggest that majority of the migrants most likely acquired their HIV-1 infection (especially CRF07_BC and CRF55_01B) in Shenzhen. This study also found that the prevalence of CRF01_AE and B had decreased, while the prevalence of CRF07_BC and CRF55_01B had rapidly increased among individuals reporting MSM risk in Shenzhen, from 2005 to 2012 (Fig. 1). In particular, since 2010, CRF07_BC replaced CRF01_AE as the predominate HIV-1 subtype among MSM in Shenzhen, and had significantly higher prevalence among MSM than those reporting HTS and IDU risks (Fig. 3). Although an increased prevalence of CRF07_BC among MSM was also observed in other regions in recent years^12^, it is the first time that the prevalence of CRF07_BC was observed to exceed CRF01_AE among MSM (Fig. 1). Interestingly, the prevalence of CRF07_BC and CRF01_AE subtypes among men reporting HTS risk was intermediate between individuals reporting MSM risk and females reporting HTS risk (Fig. 3), suggesting that some of them might have not disclosed their sexual identity of MSM, and perhaps have played a role in HIV-1 transmission between HTS and MSM risk groups^34^,^35^.

It was still unclear why CRF07_BC had a very rapid expansion among MSM. We found that the two subtypes CRF07_BC (mean distance: 0.014) and CRF55_01B (0.016) had significantly lower genetic distances than those of CRF01_AE (0.039) and B (0.062) (p < 0.01, t test), indicating that the former were more genetically homogeneous than the latter. Similar observation of lower genetic distance of CRF07_BC than CRF01_AE has been reported among MSM in other studies^33^,^36^. This lower genetic distance could represent highly related and rapidly expanding transmission networks among MSM in Shenzhen and such information could be important to public health efforts^37^. To evaluate this possibility, we compared the mean genetic distance observed in the local epidemics to all sequences sampled across China. We found that the genetic distances across all subtypes except subtype B in our study cohort was significantly lower than the mean genetic distance for LANL sequences collected in China. While these results are not in contradiction with a faster growth of the CRF07_BC and CRF55_01B epidemics as previously suggested^21^, they do not support a more rapid expansion of the CRF07_BC and CRF55_01B epidemics.

This study has several limitations. First, the rapid increase of individuals reporting MSM risk among annual newly confirmed HIV cases from 3.9% in 2005 to 42.8% in 2012 could be a reporting bias due to the limited number of sequences identified from MSM available before 2008, but initial seroprevalence studies did not specifically target any risk group for testing. Second, while this study was focused on understanding the MSM risk group in Shenzhen by sequencing over half of all MSM participants, it only analyzed 11.7% and 4.4% of HIV-1 infected individuals reporting HTS and IDU risks; therefore, the comparison between MSM and other risk groups could be a biased simply due to smaller sample sizes. Third, HIV is mostly transmitted along various exposure networks, and migration influences the dynamics of these networks and the spread of the epidemic in a great extent^38^,^39^; however, we did not perform phylogenetic comparisons beyond Shenzhen. Fourth, there are six cities (Shanghai, Beijing, Guangzhou, Shenzhen, Tianjin, and Chongqing) with over 10 million people in China. These cities generally represent the most developed and most attractive areas for migrants, but Shenzhen is just one of these large cities and may not be representative of all large cities in China.

Understanding the HIV epidemic among MSM and especially migrant MSM will be important in the development and implementation of HIV prevention in China. This study clearly identified how the distribution of HIV-1 subtypes among MSM and migrant MSM has changed in Shenzhen from 2005–2012, especially in relation to CRF07_BC, which has now exceeded CRF01_AE among MSM. This study also found evidence that certain subtype subnetworks may be growing faster than others, e.g. CRF07_BC and CRF55_01B vs. CRF01_AE and B. Such information could be important in any public health strategy to control HIV among MSM in China.

## Methods

### Ethic Statement

This study was approved by the Medical Ethics Committee of Shenzhen Center for Disease Control and Prevention and all experiments were performed in accordance with relevant guidelines and regulations. Demographic and epidemiologic data and clinical data were collected from standardized interviews. Informed consent was obtained from each participant.

### Study population and data collection

A total of 1,862 individuals reporting MSM risk were diagnosed as HIV-1 seropositive in Shenzhen city between 2005–2012. A total of 1,280 (68.7%) valid blood samples were collected from these HIV sero-positive individuals by their follow-up visit, and subjected to HIV genomic amplification and sequencing. From these 1,280 samples, a total of 996 sequences of *pol* region were obtained for the analysis. An additional 403 sequences from blood samples of individuals reporting heterosexual and injection drug use (IDU) risks were also collected. The recruiting criteria also included: HIV positive, antiretroviral naive, and living in Shenzhen at time of HIV diagnosis. All blood samples were collected within 3 months of initial diagnosis of HIV infection.

### RNA extraction, cDNA synthesis and nested PCR

HIV RNA was extracted from plasma with the QIAamp Viral RNA Kit (Qiagen, Germany), according to the manufacturer’s recommendations. About 1.3 kb HIV-1 PR-RT region (2147-3462 nt in HXB2) was amplified using RT-nested-PCR using the PrimeScript™ one step RT-PCR kit (TAKARA). Amplified products were confirmed by 1.0% agarose gel electrophoresis. After purification, the products were sequenced using primers PRO-1, RT-20, RTA and RTB, as previously described^40^. The information of the primer pairs used in this study is shown in the supplementary Table S2.

### HIV-1 subtyping

All sequences were first subjected to HIV BLAST search to exclude the possibility of contamination by laboratory-adapted strains. Sequences were then aligned using MUSCLE implemented in MEGA 6.0^41^. If there were identical sequences, the RT-nested-PCR and sequencing were re-performed to best exclude the cross-contamination between samples.

First, sequences were determined using the online REGA HIV-1 subtyping tool^42^. Second, sequences were aligned with HIV-1 reference sequences from HIV LANL^20^, and then a maximum likelihood (ML) tree was constructed using MEGA 6.0 with 100 bootstrap replicates under the model of general time reversible with gamma distributed with invariant sites (GTR + G + I, 5 discrete gamma categories)^41^. The final subtype of each sequence was based on the ML tree. For the sequences that were identified as recombinants by REGA or did not cluster within the clades of known HIV-1 subtypes or CRFs in ML tree, bootscanning analyses were performed to determine the recombination patterns using Simplot 3.5.1^43^. The recombinants were further confirmed using Recombinant Identification Program (RIP) implemented in HIV sequence Database (http://www.hiv.lanl.gov/content/sequence/RIP/RIP.html)^44^. The overall mean genetic distance within each subtype was estimated using MEGA 6.0 with p-distance method considering transitions and transversions.

### Statistical analyses

The mean Tamura-Nei 93 genetic distances within each subtype were computed using MEGA 6.0^41^,^45^, The differences in genetic distance between subtypes were assessed by comparing the pairwise distances between two subtypes using the T-test. A mixed-effects linear regression model was used to analyze the dynamic change of HIV subtype prevalence overtime.

### Nucleotide sequence accession numbers

Most of the CRF55_01B sequences had been submitted to GenBank previously under accession numbers of KF857358-KF857460^18^. All other sequences reported in this paper have been submitted to GenBank and accession numbers are KT378642-KT379957.

## Additional Information

**How to cite this article**: Zhao, J. *et al*. The dynamics of the HIV epidemic among men who have sex with men (MSM) from 2005 to 2012 in Shenzhen, China. *Sci. Rep.* **6**, 28703; doi: 10.1038/srep28703 (2016).

## Supplementary Material

Supplementary Information

## Figures and Tables

**Table 1:** Socio-demographic characteristics of the 996 HIV-1 infected individuals reporting MSM risk in Shenzhen from 2005–2012.

Characteristics	Number	Percentage (%)
Age (years old)		
20–25	69	6.9
26–35	543	54.5
36–45	295	29.6
46–55	70	7.0
56–65	12	1.2
>65	7	0.7
Marital status		
Single	740	74.3
Married	183	18.4
Divorced, separated or widowed	52	5.2
NA	21	2.1
Nationality		
China (including one from Kong Kong)	995	99.9
Other countries	1	0.1
Census registration		
Local (permanent) resident	97	9.7
Temporary resident*	605	60.7
Floating population*	294	29.5
Occupation		
Commercial service	326	32.7
Worker	212	21.3
Official	74	7.4
Catering services	36	3.6
Migrant laborer	20	2.0
Teacher	9	0.9
Health worker	7	0.7
Baby-sitter	1	0.1
Other occupations	127	12.8
Farmer	1	0.1
Student	10	1.0
Retired	5	0.5
Unemployment	86	8.6
NA	82	8.2
Education		
Junior high school or lower	313	31.4
Senior high school	384	38.6
College	295	29.6
NA	4	0.4
Ethnic background		
Han	955	95.9
Zhuang	13	1.3
Miao	7	0.7
Tujia	4	0.4
Mongol#	4	0.4
Hui	3	0.3
Uyghur	2	0.2
Korean	1	0.1
Tibetan	1	0.1
Dongxiang	1	0.1
Other	5	0.5
Total	996	

NA, not available.

^#^including one non-Chinese resident.

*Temporary resident indicates the individuals who have household registrations in other regions, and have stayed in Shenzhen city more than six months. Floating population indicates the individuals who have household registrations in other regions, and have stayed in Shenzhen less than six months. The places of household registration are shown in supplementary Table S1.
