# Supplementary material for: The dynamics of the HIV epidemic among men who have sex with men (MSM) from 2005 to 2012 in Shenzhen, China
Source: Sci Rep. 2016 Jun 29;6:28703. doi: 10.1038/srep28703 (PMC4926087; doi:10.1038/srep28703)
Supplement: Supplementary Information [file srep28703-s1.pdf]

SUPPLEMENTARY MATERIAL

The dynamics of the HIV epidemic among men who have sex with men (MSM) from 2005 to 2012 in Shenzhen, China

Running title: Dynamic of the HIV Epidemic among MSM in Shenzhen

Jin Zhao[1, †], Lin Chen[1, †], Antoine Chaillon[3, 4, †], Chenli Zheng[1], Wende Cai[1], Zhengrong Yang[1], Guilian Li[1], Yongxia Gan[1], Xiaohui Wang[1], Yihong Hu[2], Ping Zhong[5], Chiyu Zhang[2, 3, 4, *], Davey M. Smith[3, 4, *]

Affiliation

1. Shenzhen Center for Disease Control and Prevention, Shenzhen, China
2. Pathogen Diagnostic Center, Institut Pasteur of Shanghai, Chinese Academy of Sciences, Shanghai, China
3. University of California San Diego, La Jolla, California, USA
4. Veterans Affairs Healthcare System San Diego, San Diego, California, USA
5. Shanghai Municipal Center for Disease Control and Prevention, Shanghai, China

* Corresponding author: Davey M. Smith, University of California San Diego, 9500 Gilman Dr. (mail code 0679), La Jolla, CA 92093 USA, Tel: 858-642-1620, Fax: 858-552-7446, Email: davey@ucsd.edu

* Alternate corresponding author: Chiyu Zhang, Pathogen Diagnostic Center, Institut Pasteur of Shanghai, Chinese Academy of Sciences, 411 Hefei Road,

Shanghai 200025,China, Tel: 86-21-5492-3011, Fax: 86-21-5492-3044 , Email:

zhangcy1999@ips.ac.cn

† J. Z., L. C. and A. C. contributed equally to this work.

Table S1. The places of household registration (*Hukou*) of 996 HIV-1 infected individuals with MSM risk in Shenzhen.

Places of household registration (number, %)	Provinces of China/other countries	Abbreviation	Number	Percentage (%)
Northeastern China (93, 9.3)	Beijing	BJ	4	0.4
	Hebei	HeB	18	1.8
	Heilongjiang	HLJ	31	3.1
	Inner Mongolia	IM	2	0.2
	Jilin	JL	13	1.3
	Liaoning	LN	17	1.7
	Shanxi	SX	8	0.8
Northwestern China (45, 4.5)	Gansu	GS	10	1.0
	Ningxia	NX	2	0.2
	Qinghai	QH	1	0.1
	Shannxi	S'X	28	2.8
	Xinjiang	XJ	4	0.4
Central China (335, 33.6)	Henan	HeN	54	5.4
	Hubei	HuB	98	9.8
	Hunan	HuN	126	12.7
	Jiangxi	JX	57	5.7
Eastern China (72, 7.2)	Anhui	AH	19	1.9
	Fujian	FJ	10	1.0
	Jiangsu	JS	11	1.1
	Shandong	SD	18	1.8

	Shanghai	SH	5	0.5
	Zhejiang	ZJ	9	0.9
Southwestern China (214, 21.5)	Chongqing	CQ	29	2.9
	Guangxi	GX	52	5.2
	Guizhou	GZ	23	2.3
	Sichuan	SC	102	10.2
	Yunnan	YN	7	0.7
	Tibet	XZ	1	0.1
Southern China (235, 23.6)	Guangdong	GD	231	23.2
	Hainan	HaiN	3	0.3
	Hong Kong	HK	1	0.1
Other countries (1, 0.1)	Philippines	PH	1	0.1
NA	NA	NA	1	0.1
Total			996	100.0

NA, not available.

Table S2. Information of the primer pairs used in this study.

Reactions	Primer	Sequence (5'-3')	Location in HXB2
1st round PCR	MAW-26	TGGAAATGTGGAAAGGAAGGAC	2032-2050
	RT-21	CTGTATTTCTGCTATTAAGTCTTTTGA	3513-3539
2nd round PCR	PRO-1*	CAGAGCCAACAGCCCCACCA	2147-2166
	RT-20*	CTGCCAGTTCTAGCTCTGCTTC	3441-3462
Sequencing reaction	RTA	GTTGACTCAGATTGGTTGCAC	2519-2539
	RTB	CCTAGTATAAACAATGAGACAC	2946-2967

* The primers for the 2nd round PCR amplification were also used for the sequencing reaction.

Table S3. Mean pairwise genetic distance of Subtype B and CRF 07_BC CRF08_BC and CRF01_AE

HIV-1 Subtypes	HIV LANL Database of sequences from CHina*	Shenzhen study	P value
CRF07_BC	0.033 (n= 153 seq)	0.014 (n= 389 seq)	<0.01
CRF08_BC	0.031 (n= 212 seq)	0.026 (n= 29 seq)	<0.01
CRF01_AE	0.047 (n= 999 seq)	0.039 (n= 350 seq)	<0.01
Subtype B	0.045 (n= 422 seq)	0.062 (n= 101seq)	<0.01

*HIV *pol* sequences from China were downloaded from the HIV LANL database (http://www.hiv.lanl.gov) on April 20, 2016. Because the sequences from Shenzhen were not released from the database, they were not included in the downloaded sequences.

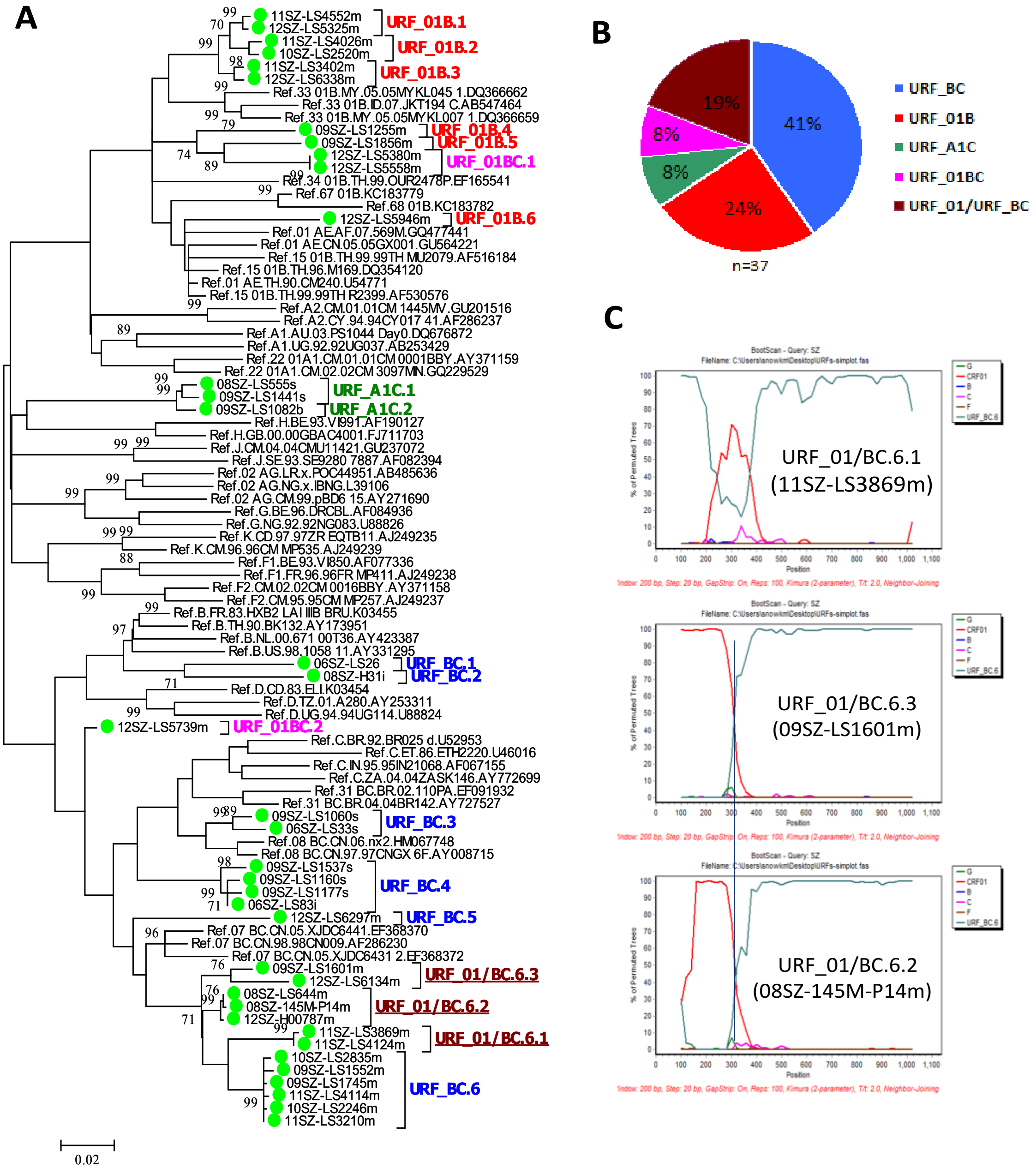

Fig. S1. HIV-1 URFs identified in Shenzhen from 2006 to 2012. **A**, the ML tree of the recombinants; **B**, proportion of various recombination forms; **C**, the bootscan plots of the 2nd generation URFs between CRF01_AE and URF_BC.6. The recombinants were confirmed by Simplot 3.5.1 and RIP, and the results obtained by both methods were consistent. For the 2nd generation URFs, only the bootscan plots by Simplot 3.5.1 are shown. There were 37 recombinants identified from 1399 HIV-1 infected individuals, including 26 from 996 MSM. These recombinant forms included 15 (40.5%) URFs_BC, 9 (24.3%) URFs_01/B, 3 (8.1%) URFs_A1/C, 3 (8.1%) URFs_01BC and 7 (18.9%) URF_01/URF_BC (2nd generation of URFs between CRF01_AE and

URFs_BC). These recombinants formed 19 lineages, including URF_BC.1-URF_BC.6, URF_01B.1-URF_01B.6, URF_A1C.1-URF_A1C.2, URF_01BC.1-URF_01BC.2, and URF_01/URF_BC.6.1- URF_01/URF_BC.6.3. The last characters “m”, “s”, “i” and “b” at the sequence name indicate the sequences from individuals reporting MSM, heterosexual, IDU and bisexual risks, respectively.

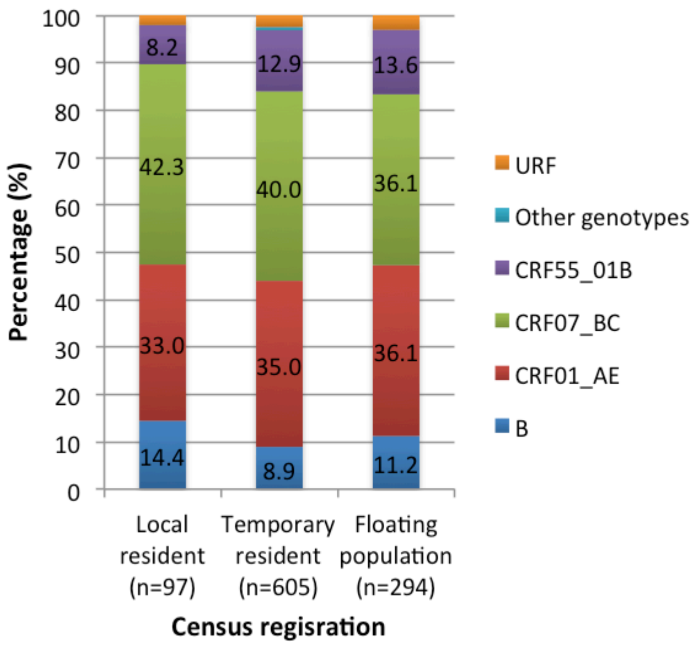

Fig. S2. HIV-1 genotype distributions by census registration (*Hukou*).
